# Defining Oligometastatic Disease in the New Era of PSMA-PET Imaging for Primary Staging of Prostate Cancer

**DOI:** 10.3390/cancers14143302

**Published:** 2022-07-06

**Authors:** Samuel J. Galgano, Andrew M. McDonald, Janelle T. West, Soroush Rais-Bahrami

**Affiliations:** 1 Department of Radiology, University of Alabama at Birmingham, Birmingham, AL 35294, USA; samuelgalgano@uabmc.edu (S.J.G.); janellewest@uabmc.edu (J.T.W.); 2 O’Neal Comprehensive Cancer Center, University of Alabama at Birmingham, Birmingham, AL 35294, USA; ammcdonald@uabmc.edu; 3 Department of Radiation Oncology, University of Alabama at Birmingham, Birmingham, AL 35294, USA; 4 Department of Urology, University of Alabama at Birmingham, Birmingham, AL 35294, USA

**Keywords:** prostatic adenocarcinoma, cancer staging, lymph node metastases, prostate specific membrane antigen

## Abstract

**Simple Summary:**

Oligometastatic prostate cancer has classically been defined as a small volume of prostate cancer spread as defined by imaging using CT, MRI, and bone scans. With development and integration of more sensitive functional imaging including PSMA-PET imaging more oligometastatic disease will be detected than was found with conventional imaging techniques. We are currently lacking data directing the best treatment course for these oligometastatic prostate cancer cases found on PET imaging who would have been defined as localized disease prior to PET imaging. Herein, we discuss the concept of stage migration and discuss current problems and challenges with the current definition of oligometastatic disease as imaging modalities have progressed in our field.

**Abstract:**

Oligometastatic prostate cancer has traditionally been defined in the literature as a limited number of metastatic lesions (either to soft tissue or bone), typically based on findings seen on CT, MRI, and skeletal scintigraphy. Although definitions have varied among research studies, many important clinical trials have documented effective treatments and prognostication in patients with oligometastatic prostate cancer. In current clinical practice, prostate-specific membrane antigen (PSMA)-PET/CT is increasingly utilized for the initial staging of high-risk patients and, in many cases, detecting metastases that would have otherwise been undetected with conventional staging imaging. Thus, patients with presumed localized and/or oligometastatic prostate cancer undergo stage migration based on more novel molecular imaging. As a result, it is challenging to apply the data from the era before widespread PET utilization to current clinical practice and to relate current trials using PSMA-PET/CT for disease detection to older studies using conventional staging imaging alone. This manuscript aims to review the definition of oligometastatic prostate cancer, summarize important studies utilizing both PSMA-PET/CT and conventional anatomic imaging, discuss the concept of stage migration, and discuss current problems and challenges with the current definition of oligometastatic disease.

## 1. Introduction

Prostate cancer is the most common non-cutaneous malignancy in American men, with an estimated 268,490 new cases in the United States in 2022 [1]. There is considerable variation in the overall aggressiveness of prostate cancer, with some cancers remaining indolent and others aggressively metastasizing outside the prostate. Histologically, prostate cancer is risk-stratified by the Gleason scoring system to define the grade of disease, now classified into Grade Groups ranging from 1–5, with higher scores indicative of less differentiated, more aggressive tumor histology [2]. Prostate cancer treatment is primarily dictated by the Grade Group, with the lowest Grade Group allowing for active surveillance and progressively higher-Grade Groups warranting consideration of definitive therapy. The National Comprehensive Cancer Center (NCCN) guidelines risk-stratify patients by both their Grade Group and their serum prostate-specific antigen (PSA) levels into risk categories ranging from very low-risk to very high-risk, with staging imaging indicated for patients with unfavorable-intermediate risk and above [3]. Approximately 19% of men with metastatic disease (either in lymph nodes or other organs) at their initial systemic staging are subdivided into low-volume metastatic (or oligometastatic) disease or more widespread metastatic disease based on both the number of metastases and distribution.

Given that low-volume metastatic disease is often limited to the pelvis and suitable for regional treatment and/or limited bone metastases amenable to targeted therapy if a focus extends beyond the pelvic region, the approach to the treatment of these entities differs significantly from those with systemically widespread metastases. Additionally, historical studies have found outcomes of patients with oligometastatic disease to be between those of non-metastatic and widely metastatic disease.

Newer molecular imaging agents, including prostate-specific membrane antigen (PSMA)-PET/CT and fluciclovine-PET/CT, have substantially improved the detection of metastatic disease that otherwise would have gone undetected on conventional imaging [4]. This phenomenon is known as stage migration (or the Will Rogers effect), where a patient who appeared to have cancer limited to the prostate on conventional imaging is found to have extraprostatic malignancy on PET imaging, resulting in changes in prognosis and outcome [5]. Thus, there is uncertainty in the current practice of how studies performed prior to prostate cancer-directed PET imaging can be translated into the era of the improved sensitivity of targeted molecular imaging of prostate cancer. The purpose of this manuscript is to outline the historical definition of oligometastatic prostate cancer, discuss relevant studies prior to the widespread use of PET imaging, and discuss how PET imaging of prostate cancer has led to an evolving definition and treatment of patients harboring oligometastatic prostate cancer.

## 2. Historical Definition of Oligometastatic Disease and Relevant Studies

Generally speaking, the term oligometastatic disease is defined as either a limited number and/or volume of metastatic lesions and applies to a number of different malignancies [6,7,8]. Typically, studies defining and evaluating outcomes of patients with oligometastatic disease use their own study-specific definition of oligometastatic disease, which often ranges from 1–5 metastatic lesions. For patients with a prostate cancer diagnosis, the two most commonly utilized and widely referenced definitions of oligometastatic disease come from the CHAARTED and LATITUDE clinical trials extrapolating from the trial definitions of “low volume” disease burden or “non-high-risk” clinical criteria, respectively [9,10]. A study evaluating the overall metastatic disease burden comparing definitions between these trials found excellent agreement and significant prognostic value from these definitions [11]. In the CHAARTED trial, patients were stratified into low volume hormone-sensitive and high volume hormone-sensitive (defined as the presence of visceral metastases or ≥4 bone lesions with ≥1 beyond the vertebral bodies and pelvis) metastatic disease, with both groups demonstrating improved overall survival with the addition of docetaxel to androgen deprivation therapy alone [10]. The LATITUDE trial demonstrated a benefit in overall survival and radiographic progression-free survival in men with newly diagnosed, metastatic, castration-sensitive prostate cancer treated with the addition of abiraterone and prednisone to androgen deprivation therapy and utilized a different definition with “high-risk” patients demonstrating at least two of the following criteria: Gleason score of ≥8, at least three bone lesions, and presence of measurable visceral, non-nodal, soft tissue metastases [9].

As with many critical clinical trials that have taken place before the widespread use of emerging PET radiotracers for prostate cancer staging, these definitions rely primarily on findings as seen on CT, MRI, and ^99m^Tc-methyl diphosphonate (MDP) skeletal scintigraphy. Through these trials (and others, including the STAMPEDE and HORRAD trials), many important conclusions were made regarding the prognosis and treatment of patients with oligometastatic prostate cancer at the time, often utilizing a combination of systemic therapy, targeted radiotherapy, and/or surgical resection with varying definitions of oligometastatic disease [12,13,14,15,16,17,18,19,20,21,22]. However, as the utilization of PET-based molecular imaging for prostate cancer staging has increased, there is increasing uncertainty on how to apply the conclusions of these important clinical trials, performed in the past relying on conventional staging imaging modalities to disease detected through higher sensitivity, more novel PET imaging.

## 3. PET Radiotracers Used for Prostate Cancer

Table 1 lists the current FDA-approved radiotracers that can potentially be utilized for molecular imaging of prostate cancer. ^18^F-fluorodeoxyglucose (FDG) is not routinely used for imaging prostate cancer, as lower-grade prostatic adenocarcinoma typically does not demonstrate significant uptake [23]. ^18^F-fluciclovine, and ^11^C-choline are only approved for use in biochemically recurrent prostate cancer and not initial staging due to challenges in differentiating benign prostatic hyperplasia nodules from prostate cancer foci in an intact, untreated prostate gland [24,25]. However, emerging data suggest that these radiotracers may have value in the initial staging of select patient populations, and some of the limitations may be overcome by using PET/MRI [26,27,28,29]. ^18^F-sodium fluoride (NaF) is a radiotracer that physiologically functions similarly to ^99m^Tc-MDP and demonstrates accumulation at sites of increased bone turnover (such as sclerotic osseous metastases) [30]. At staging, ^18^F-NaF can be used in lieu of skeletal scintigraphy for bone-specific imaging, but at a higher cost and still necessitates additional imaging for staging of non-osseous metastatic disease.

PSMA is a transmembrane glycoprotein that is overexpressed on the cell surface of prostate cancer cells [31]. The PSMA transmembrane protein has long been a target of molecular imaging for patients with prostate cancer, first utilizing ^111^In-capromab pendetide gamma camera imaging and now PSMA-targeted PET radiotracers. Several PSMA PET radiotracers are currently FDA-approved for use in the initial staging of high-risk patients, and these tracers can be radiolabeled with both ^68^Ga and ^18^F. PSMA PET radiotracers allow for whole-body staging in a single imaging session. The current National Comprehensive Cancer Network guidelines suggest that PSMA PET can be the initial imaging modality used for prostate cancer staging [3]. Although no large head-to-head comparisons of PSMA radiotracers have been performed, these tracers all function very similarly to one another and offer superior diagnostic performance compared to other PET radiotracers and conventional anatomic staging imaging in the detection of extraprostatic metastatic deposits [32,33,34,35,36].

## 4. Studies Integrating PET into Diagnosis and Treatment of Oligometastatic Disease

Given the increasing data on improved sensitivity and specificity of PET radiotracers for improving staging of prostate cancer when compared to conventional imaging, modern-day clinical practice and clinical trials have begun to utilize PET/CT for staging imaging over CT, MRI, and skeletal scintigraphy, adding a recognized stage migration with earlier detection of regional or metastatic prostate cancer spread [36,37]. In particular, multiple studies evaluating the use of stereotactic radiation or other focal therapy for the treatment of oligometastatic have relied heavily on PET/CT (with various radiotracers) due to a better selection of patients who are truly oligometastatic [38,39]. An early study evaluating the use of ^11^C-choline PET/CT demonstrated that this approach could produce a useful single diagnostic exam in the evaluation of patients with moderate to high-risk prostate cancer with the potential of metastatic spread and can reliably rule out lymph node and distant metastases and establish eligibility for pelvic radiotherapy [40]. Another study found that the image-guided ablation of oligometastases identified by ^11^C-choline PET/CT offered acceptable local tumor control rates and may allow for a delay of ADT initiation [41].

The most recent work has utilized various PSMA radiotracers for guiding staging and treatments in the setting of oligometastatic prostate cancer. The proPSMA trial was a prospective, multicenter trial in patients with newly diagnosed high-risk prostate cancer, found that PSMA PET/CT demonstrated 27% greater accuracy than conventional imaging and was a suitable replacement to current conventional imaging methods [42]. The ORIOLE trial utilized ^18^F-DCFPyL PET/CT in a phase II randomized trial of observation versus stereotactic ablation radiation in oligometastatic prostate cancer [43]. All treating physicians in this study were blinded to the PET/CT data during treatment planning, and if lesions identified on DCFPyL PET/CT were not treated, these patients demonstrated significantly decreased composite progression-free survival and distant metastasis-free survival [43]. Additional studies utilizing other PSMA PET radiotracers in guiding radiotherapy have also produced similar benefits [44,45,46].

## 5. Current Status and Future Directions

It is without question that PSMA-PET/CT has significantly improved the staging and restaging of patients with prostate cancer when compared to conventional anatomic imaging. Given this improved diagnostic performance, significant uncertainties exist in clinical and research settings on what constitutes oligometastatic disease [47,48]. As a result, various international groups have issued several consensus opinions to establish consensus definitions, which will aid in ongoing clinical trials and facilitate more uniform clinical care [49,50]. It is critical to acknowledge the limitations of using data derived from decades of conventional imaging for the purposes of prostate cancer staging in the new era of PSMA PET imaging with a resultant stage migration effect. However, recognizing the robust body of data that does exist to define the improvements of clinical outcomes with treatment for each of the various stages defined with conventional imaging, this is the foundation of determining future treatment algorithms regardless of imaging modality used in the modern era until there is additional data from clinical trials and practice case series uniformly using PET imaging for staging. That being said, it is clear that patients who were previously thought to be nonmetastatic based on conventional imaging will be reclassified and migrate to an oligometastatic stage of their prostate cancer on the basis of PET imaging alone. 

The primary question in regard to this stage migration to a higher stage classification for some oligometastatic prostate cancer patients is how to apply data from the era of conventional imaging to an era where molecular imaging improves detection of disease which is otherwise occult on conventional staging techniques. Some proportion of men with oligometastatic disease on conventional imaging who were included in early trials (such as LATITUDE and CHAARTED) would likely have been upstaged to a more widespread metastatic disease definition if PET imaging had been utilized at the time.

Conversely, it is challenging to relate emerging data utilizing PET imaging to historical data on the overall survival of patients with oligometastatic prostate cancer [51]. Ultimately, while we now can better understand and visualize the burden of metastatic disease, patient prognosis remains unclear. We recognize that many of the necessary trials to redefine the optimal treatment algorithms in the era of PSMA PET-derived staging definitions would require years of patient accrual and several years of follow-up to redefine or validate findings of prior trials using conventional imaging modalities alone. For the current dynamic in play, we would recommend that clinical trials and studies in the future could consider statistical analyses based on both conventional staging imaging as well as PET imaging to provide a bridge between historical data predating routine PET imaging and current clinical practice patterns, further allowing translation and use of clinical trials with a longer follow-up that only used conventional imaging in the past.

## 6. Conclusions

While we recognize the optimized sensitivity of extraprostatic prostate cancer foci detection with PSMA PET imaging resulting in a stage migration in the initial diagnosis and staging of oligometastatic prostate cancer, we also know that the management of these patients is as uncertain as ever before. As such, more work is needed to truly establish the clinical impact of improved disease detection through PSMA-PET/CT.

## Figures and Tables

**Table 1:** List of FDA-Approved Radiotracers for Prostate Cancer.

Radiotracer	Indicated for Initial Staging
^18^F-FDG	No
^18^F-sodium fluoride	Yes
^11^C-choline	No
^18^F-PSMA/^68^Ga-PSMA	Yes
^18^F-fluciclovine	No

## Data Availability

Data used for this literature review are publicly available via the cited published literature.
